# Tandemly Integrated HPV16 Can Form a Brd4-Dependent Super-Enhancer-Like Element That Drives Transcription of Viral Oncogenes

**DOI:** 10.1128/mBio.01446-16

**Published:** 2016-09-13

**Authors:** Katharine E. Dooley, Alix Warburton, Alison A. McBride

**Affiliations:** Laboratory of Viral Diseases, National Institute of Allergy and Infectious Diseases, National Institutes of Health, Bethesda, Maryland, USA

## Abstract

In cancer cells associated with human papillomavirus (HPV) infections, the viral genome is very often found integrated into the cellular genome. The viral oncogenes E6 and E7 are transcribed from the viral promoter, and integration events that alter transcriptional regulation of this promoter contribute to carcinogenic progression. In this study, we detected highly enriched binding of the super-enhancer markers Brd4, MED1, and H3K27ac, visible as a prominent nuclear focus by immunofluorescence, at the tandemly integrated copies of HPV16 in cells of the cervical neoplasia cell line W12 subclone 20861. Tumor cells are often addicted to super-enhancer-driven oncogenes and are particularly sensitive to disruption of transcription factor binding to the enhancers. Treatment of 20861 cells with bromodomain inhibitors displaced Brd4 from the HPV integration site, greatly decreased E6/E7 transcription, and inhibited cellular proliferation. Thus, Brd4 activates viral transcription at this integration site, and strong selection for E6/E7 expression can drive the formation of a super-enhancer-like element to promote oncogenesis.

## IMPORTANCE

Oncogenic human papillomaviruses play an essential role in the development of cervical cancer, and growth of these cancer cells requires continued expression of the viral E6 and E7 oncogenes. Integration of the virus into the host genome often results in deregulation of E6 and E7 expression, which provides a selective growth advantage and increases genetic instability of infected cells. We show here that tandemly integrated copies of the viral genome can form a super-enhancer-like element that drives E6/E7 transcription. Targeted disruption of factors binding to this element decreases viral transcription and causes cell death. Thus, cancer cells that harbor integrated HPV could be targeted by therapeutics that disrupt super-enhancer function.

## INTRODUCTION

Oncogenic human papillomaviruses (HPVs) are the cause of cervical cancer, and HPV genomes, which normally replicate extrachromosomally, are often found integrated into the host genome of these cancer cells (1). Commonly, either a single viral genome or multiple tandemly repeated viral genomes are integrated into the host DNA (2). The E6 and E7 oncogenes are expressed from the integrated genomes, most often as a fusion transcript expressed from the 3′ junctional copy of HPV and the adjacent cellular DNA (3). Viral genome integration promotes carcinogenesis in a number of ways, but in almost all cases, the cancer-derived cells are dependent on expression of the E6 and E7 oncogenes for continued proliferation. Integration often occurs in the HPV E2 open reading frame, which disrupts the ability of E2 to repress E6 and E7 gene expression (1). The resulting dysregulation of E6 and E7 causes disruption of cell cycle control, leading to genetic instability and carcinogenesis (4). Even when the E2 gene remains intact, methylation of E2 binding sites inhibits binding and renders the viral promoter resistant to E2 regulation (5). Integration events also occur in the E1 gene: this not only removes the downstream E2 gene, but also eliminates the growth-suppressive properties of the E1 protein (6, 7). In many cases, the E6-E7 virus-cell fusion transcript expressed from integrated DNA is more stable than the viral message, again increasing E6 and E7 levels (8). Only rarely does insertional mutagenesis result in modified expression of cellular oncogenes or tumor suppressors (9). In this study, we identify an additional mechanism of E6 and E7 oncogene upregulation. We show that multiple tandem copies of integrated HPV16 can act as a Brd4-dependent super-enhancer-like element that drives transcription of the E6 and E7 oncogenes.

Brd4, a double bromodomain and extraterminal domain (BET) protein, plays an essential role in cellular transcription by binding acetylated histones and recruiting positive transcriptional complexes to promoters. Brd4 also plays an important role in transcriptional regulation and replication of papillomaviruses (reviewed in reference 10). The viral E2 protein binds to Brd4 and stabilizes its association with chromatin (11, 12). However, in the context of the HPV early promoter, Brd4 and E2 primarily repress viral transcription (13–15), in part because E2 interacts with the C-terminal domain (CTD) of Brd4 and blocks the formation of the Brd4-pTEFb complex (16). In this way, Brd4 commonly acts as an E2-dependent repressor of E6 and E7 oncogene transcription.

Super-enhancers have been defined as the spatial clustering of large groups of traditional enhancers that control genes responsible for cell identity (17–19). Super-enhancers are also associated with the expression of oncogenes; for example, a super-enhancer often drives the *MYC* gene in multiple myeloma (20). Binding of transcription factors, cofactors, and chromatin regulators is enriched at super-enhancers; specifically, super-enhancers can be identified by a high density of mediator, acetylated H3K27, and Brd4 (18, 19). Super-enhancers are particularly sensitive to disruption of transcription factor binding. Super-enhancers often drive gene products to which the cells are “addicted,” which has great therapeutic potential as it allows targeted disruption of gene expression from super-enhancer-regulated disease-causing genes (20). Here we show that there is highly enriched binding of the super-enhancer marker mediator (MED1), acetylated H3K27, and Brd4 at the integrated locus of HPV16 in 20861 cells. Furthermore, we show that disruption of Brd4 binding to this locus reduces HPV16 oncogene expression, resulting in greatly decreased cellular proliferation and induction of senescence.

## RESULTS

### Brd4 is highly enriched at the locus of HPV integration in W12 20861 cells.

**FIG 1:**
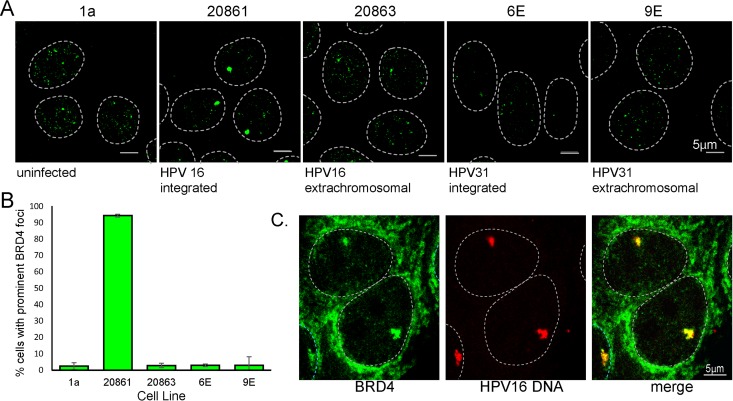
Brd4 forms a prominent focus at the integrated HPV16 locus in W12 20861 cells. (A) Brd4 was detected in 1a, 20861, 20863, 6E, and 9E cervical cells by indirect immunofluorescence with the 8H2 Brd4 antibody (green). Dotted lines show the outline of the nucleus. The data and images shown were obtained from z-stacks of the entire cell combined using maximum projection. (B) The cell lines shown in panel A were examined for prominent Brd4 foci. Error bars represent the standard deviation (SD) from three independent experiments (≥57 cells per experiment). (C) Viral DNA was detected by FISH with an HPV16 probe (red) and Brd4 with the MCB2 antibody (green). Brd4 foci were examined for colocalization with HPV16 foci: 94% ± 5% of Brd4 foci were positive for HPV16 (mean ± SD; *n =* 3). The dotted lines show the outline of the nucleus.

W12 cells were originally derived from an HPV16-positive CIN1 (cervical intraepithelial neoplasia grade 1) lesion (21). The original cells consisted of a mixed population of cells containing either extrachromosomal or integrated HPV16 genomes, but subsequently a series of clonal cell lines were derived that stably maintained the viral genome extrachromosomally or as integrants in the host genome (2). Analysis of Brd4 by immunofluorescence (IF) in the W12 subclone 20861 revealed a large and prominent focus of Brd4 signal in the nucleus of virtually every cell (Fig. 1A). This subclone has approximately 30 copies of the HPV16 genome integrated in tandem at a single locus (2), raising the possibility that the observed Brd4 focus corresponded to the HPV16 integration site. In comparison, as shown in Fig. 1A and B, no similar conspicuous focus of Brd4 could be detected in HPV-negative 1A keratinocytes, the 20863 subclone of W12 (with extrachromosomal HPV16 genomes), the 6E subclone of CIN612 cells (with similarly tandemly integrated copies of HPV31), and the 9E subclone of CIN612 cells (with extrachromosomal HPV31 genomes). To prove that the prominent Brd4 focus corresponded to the HPV16 integration site, immunofluorescent *in situ* hybridization (immuno-FISH) was performed to simultaneously detect viral DNA and the Brd4 protein. As shown in Fig. 1C, the Brd4 focus overlaps the HPV integration locus in about 94% cells.

### Brd4 binding at the HPV integration locus is disrupted by BET inhibitors.

**FIG 2:**
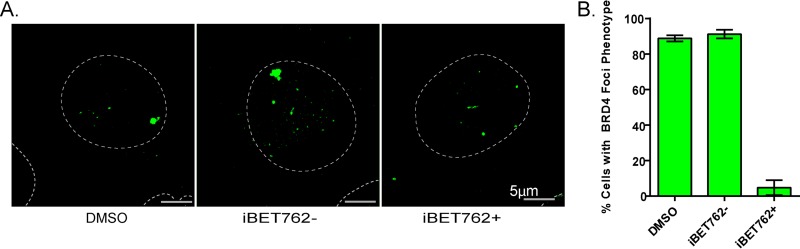
iBET treatment disrupts the prominent Brd4 focus in 20861 cells. (A) 20861 cells were treated with either dimethyl sulfoxide (DMSO), 1 µM iBET762+, or 1 µM iBET762− for 22 h (with medium refreshed 4 h before fixation). Cells were fixed, and Brd4 was detected by immunofluorescence with the 8H2 antibody. The data and images shown were obtained from a maximal projection of z-stacks encompassing the entire cell. Dotted lines show the outline of the nucleus. (B) A minimum of 61 cells per experiment were examined for Brd4 foci. Error bars represent SD from three independent experiments.

The bromodomains of Brd4 interact with chromatin through acetylated histone tails (22). Several inhibitors, which mimic acetylated lysine, have been developed to disrupt the binding of BET bromodomains to chromatin (23, 24). To show that Brd4 binding to the HPV integration locus was mediated through acetylated histone tails, 20861 cells were treated with 1 µM iBET762 (or controls) for 22 h before detection of Brd4 by immunofluorescence. As shown in Fig. 2, detectable Brd4 binding at the HPV integration locus was reduced to less than 5% of cells.

### The HPV integration locus is not a site of genetic instability.

Ustav et al. have shown that integrated HPV can undergo multiple rounds of replication in the presence of the E1 and E2 replication proteins, and this induces the recruitment of markers of the DNA damage response (25). We also find that Brd4 can be recruited to HPV integration sites, but only in the presence of E1 and E2 (unpublished data). Integrated HPV genomes generally do not express the E1 and E2 proteins, but nevertheless we examined the 20861 Brd4 focus/HPV integration site for the presence of γ-H2AX and Rad51, markers of DNA damage and homologous recombination. Only rare (3 to 4%) cells showed any colocalization of Brd4 with these markers (see Fig. S1 in the supplemental material). Therefore, Brd4 binding is not due to genetic instability of the HPV integration locus.

### Brd4 does not bind to the HPV integration locus throughout mitosis.

We have also shown that Brd4 and HPV1 E2 bind to host chromatin at regions associated with common fragile sites of the host genome that have been designated PEB-BLOCs (for *p*ersistent *E*2 and Brd4 *b*road *l*ocalizations of *c*hromatin) (26). Notably, Brd4 binds PEB-BLOCs throughout the cell cycle, in contrast to its interaction with cellular promoter regions from which it is displaced in mitosis (26). Therefore, we analyzed the presence of the prominent speckle of Brd4 in 20861 cells and found that it does not persist strongly throughout mitosis (see Fig. S2 in the supplemental material). We conclude that Brd4 binding to the HPV integration site in 20861 cells is not related to PEB-BLOCs.

### Brd4 activates HPV16 transcription in 20861 cells.

**FIG 3:**
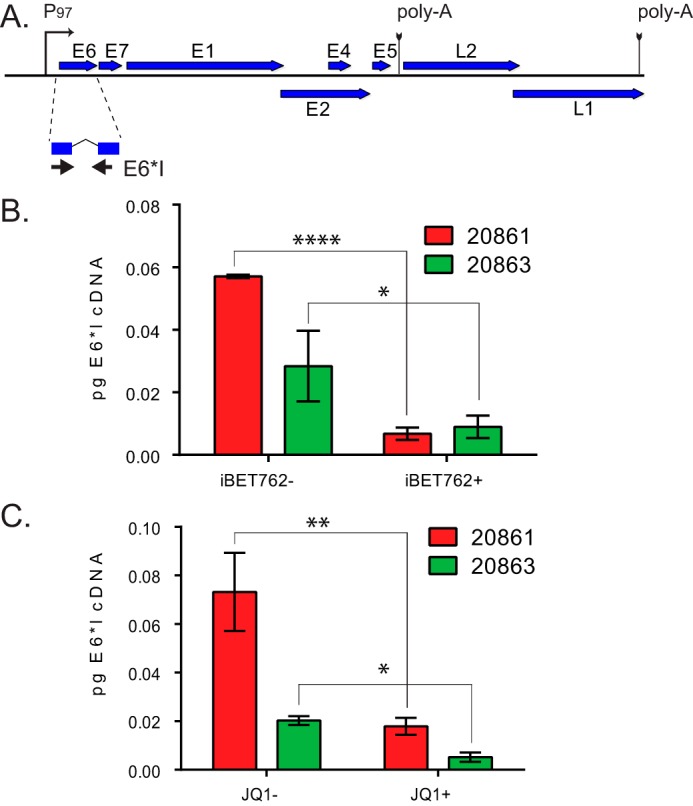
Brd4 is an activator of HPV 16 transcription at the integrated HPV16 locus in 20861 cells. (A) Map of linearized HPV16 genome with position of the E6*I transcript and location of primers used to detect E6*I cDNA. (B and C) 20861 and 20863 cells were treated with DMSO, 1 µM iBET762+, or 1 µM iBET762− (B) or DMSO, 250 nM JQ1+, or 250 nM JQ1− (A) for 44 h (with fresh medium/inhibitors added after 24 h and for the last 4 h), and HPV16 E6*I transcription levels were determined by real-time qPCR. The amount of E6*I cDNA was determined using a standard curve and normalized to 28S rRNA. For panel B, results are from three independent experiments. For panel C, the results shown are from 3 technical replicates, but at least 3 additional independent experiments with similar, but not identical, inhibitor concentrations and timings were conducted with similar results. Error bars represent SD. An unpaired Student’s *t* test was used to determine statistical significance between treatments. *, *P* < 0.05; **, *P* < 0.005; ****, *P* < 0.0001.

Based on the findings described above, it seemed most likely that Brd4 was regulating transcription at the integrated HPV locus. Brd4 has been shown to both activate and repress transcription of the HPV early promoter at integrated loci (13, 16, 27). To analyze whether Brd4 regulated viral transcription from the integrated locus of HPV16, 20861 cells were treated with the bromodomain inhibitor iBET762 (or controls) and the early E6*I spliced transcript levels (Fig. 3A) were determined by real-time quantitative PCR (qPCR). As shown in Fig. 3B, inhibition of Brd4 binding to chromatin by iBET762 treatment dramatically suppressed viral transcription in 20861 cells. In comparison, E6*I transcription was also measured in the 20863 subclone, which contains extrachromosomal copies of HPV16. Viral transcription also decreased in 20863 cells after iBET762 treatment, indicating that Brd4 also plays an activation role in these cells. However, the fold change in transcription after BET inhibitor treatment was much greater in 20861 cells than in 20863 cells. These findings were also confirmed with a second bromodomain inhibitor, JQ1 (Fig. 3C). These data indicate that Brd4 activates viral transcription from both integrated and extrachromosomal HPV16 in these cells. However, transcription of the integrated HPV locus in 20861 cells is higher and more sensitive to disruption of Brd4 binding than in 20863 cells.

The BET inhibitors, iBET762 and JQ1, are acetyl-lysine mimics that inhibit binding of a specific class of bromodomains to acetylated histones (23, 28). Although these compounds bind with highest affinity to Brd4, they can also inhibit binding to close BET family members (29). To further prove that downregulation of HPV16 transcription was due to displacement of Brd4 from the integration locus, we downregulated Brd4 expression using small interfering RNA (siRNA) technology. As shown in Fig. S3 in the supplemental material, downregulation of Brd4 expression also decreased HPV16 E6*I expression 2.8-fold. This confirms that Brd4 activates transcription of integrated HPV16 in 20861 cells.

### pTEFb, the transcriptional pause release factor, colocalizes with Brd4 at the HPV integration locus.

**FIG 4:**
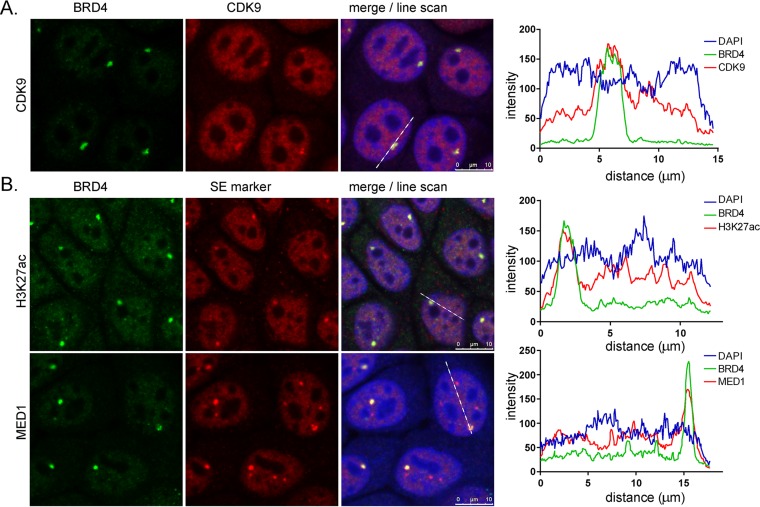
Brd4 foci colocalize with pTEFb, MED1, and CDK9. (A) Brd4 was detected by indirect immunofluorescence in 20861 cells with the 8H2 antibody and costained for CDK9. Colocalization was measured using the Leica line scan function. The dotted white line was examined for relative staining intensity an all three channels. A minimum of 73 cells were examined for colocalization in three independent replicates. CDK9 colocalized with Brd4 in 87.3% ± 4% of cells. (B) Brd4 was detected by indirect immunofluorescence in 20861 cells with the 8H2 antibody and costained for H3K27ac and MED1. Colocalization was measured using the Leica line scan function. The dotted white line was examined for relative staining intensity an all three channels. Images are from a single optical slice. A minimum of 73 cells were examined for colocalization in three independent replicates. H3K27ac colocalized with Brd4 in 98.1% ± 0.9% of cells and with MED1 in 99.6% ± 0.4% of cells.

Brd4 is classically an activator of transcription and promotes transcriptional elongation by interaction with pTEFb (30) and through a number of factors that bind the ET region (31). CDK9 is the catalytic subunit of pTEFb, and so we examined the HPV integration locus for CDK9. As shown in Fig. 4A, cyclin-dependent kinase 9 (CDK9) is highly enriched at the prominent Brd4 focus, implying that it functions to activate transcription at this locus. Colocalization of Brd4 and CDK9 suggests that Brd4 activates viral transcription from integrated HPV16 in 20861 cells by interacting with pTEFb to enhance transcriptional elongation by RNA polymerase II (Pol II).

### The HPV integration locus in 20861 cells is bound by factors related to super-enhancers.

**FIG 5:**
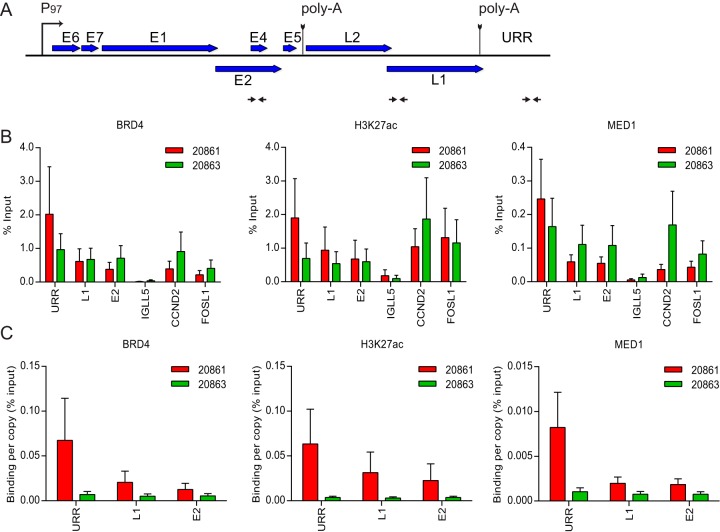
Brd4 is enriched at the integrated HPV16 genome in 20861 cells. Chromatin immunoprecipitation (ChIP) was performed in 20861 and 20863 cells using antibodies against Brd4, H3K27ac, and MED1. ChIP DNA samples were analyzed by real-time qPCR using primers against target promoters. (A) Map of linearized HPV16 genome showing primer positions (denoted by black horizontal arrows) for the upstream regulatory region (URR), L1, and E2 used for ChIP-qPCR. (B) ChIP signals were expressed as the percentage of immunoprecipitated chromatin DNA relative to the total amount of input chromatin (% Input). CCND2 and FOSL1 were included as positive controls for super-enhancer loci; IGLL5 was included as a negative control for Brd4 binding in these cells. (C) To account for variations in viral copy number between 20861 and 20863 cells, ChIP signals were expressed as binding per single-copy genome relative to percentage of input. Background signal at each locus (measured by no-antibody controls) was subtracted from corresponding ChIP signals. Average binding levels were calculated from three independent experiments. Error bars represent SD.

Super-enhancers are greatly enriched in H3K27ac and have a high density of binding of MED1 and Brd4 (17). They are also often composed of enhancers that have become tandemly repeated (18), and they often drive expression of oncogenes to which the cell is addicted (20). The latter two properties are also characteristics of many HPV integration loci. Therefore, to test whether the 20861 HPV integration locus had these additional characteristics of super-enhancers, we analyzed this region for the presence of H3K27ac and MED1. As shown in Fig. 4B, the HPV16 integration locus is greatly enriched in H3K27ac and MED1, as detected by immunofluorescence. This was further confirmed by chromatin immunoprecipitation (ChIP), which showed great enrichment for all three markers of super-enhancers over the viral genome in 20861 cells (Fig. 5). Not unexpectedly, the strongest signal in the viral genome was observed over the viral upstream regulatory region (URR). As shown in Fig. 5A, the enrichment of the super-enhancer markers exceeded that of known cancer-associated super-enhancers within the CCND2 and FOSL1 gene loci (18, 20). Enrichment was also observed over the viral URR in 20863 cells (Fig. 5A). However, displaying the data as the percentage of bound immunoprecipitated chromatin DNA relative to the total amount of input chromatin (percentage of input) does not take into consideration the wide differences in copy number among the integrated and extrachromosomal HPV genomes. Therefore, the data are shown in Fig. 5B adjusted for copy number. This shows the very high enrichment of super-enhancer markers bound to each genome copy in the HPV16 integration locus in 20861 cells. When we consider that there are 30 copies of the HPV16 genome in tandem array (2), we can appreciate the extremely strong enrichment, which corresponds to that observed by immunofluorescence (Fig. 4). Therefore, we propose that the tandem array of integrated HPV genomes can form a Brd4-dependent super-enhancer-like element in 20861 cells.

### Proliferation of 20861 cells is sensitive to disruption of Brd4 binding.

**FIG 6:**
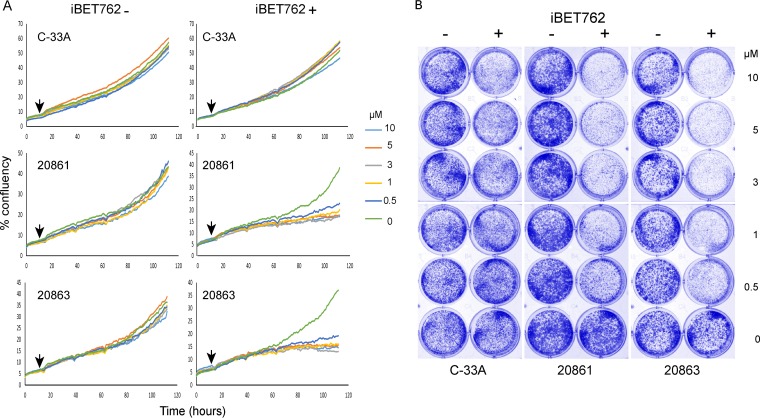
Proliferation of 20861 cells is inhibited by disruption of Brd4 binding. (A) C-33A, 20861, and 20863 cells were plated at low density, and proliferation was measured (percentage of confluence) in an Incucyte microscope. After 12 h of measurement, iBET72− or iBET72+ was added at a final concentration of 0, 0.5, 1, 3, 5, or 10 µM (time point indicated by black arrow). Growth was measured for a total of 112 h. (B) After 112 h, cells were fixed and stained with methylene blue. Five replicate growth curves were measured for 20861 and 20863 cells and three for C-33A cells. A representative experiment is shown.

Brd4 binding to the integrated HPV16 genome in 20861 cells is almost completely disrupted by bromodomain inhibitors (Fig. 2), and this disruption greatly reduces viral transcription (Fig. 3). Past studies have shown that HPV cancer cells are addicted to E6 and E7 oncogene production (32–34). Therefore, we hypothesized that the proliferation of 20861 cells would be particularly sensitive to inhibition of Brd4 binding to viral DNA. To test this, cells were plated at low density, and growth curves were determined in the presence of a titration of the bromodomain inhibitors JQ1+ and iBET72+ or their respective negative-control stereoisomers (JQ1− and iBET72−, respectively). Proliferation of 20861 cells was compared to the growth of 20863 cells, and an HPV-negative cervical cancer cell line, C-33A. As shown in Fig. 6 for iBET72+ (see Fig. S4 in the supplemental material for JQ1+), C-33A cells were relatively resistant to disruption of Brd4 binding as measured by colony growth. Both inhibitors dramatically reduced proliferation of 20861 and 20863 cells. This finding mirrors the reduction in HPV16 mRNA levels shown in Fig. 4, where both subclones showed a substantial decrease in E6 and E7 viral transcription. This confirms that growth of HPV-containing 20863 and 20861 cells is dependent on continued E6 and E7 transcription, which is regulated by Brd4 expression and can be modulated by BET inhibitors.

### Gene expression analysis confirms that W12 cells undergo senescence in response to decreased E6 and E7 gene expression.

Using amplification of papillomavirus oncogene transcripts (APOT), we have determined that in 20861 cells, HPV16 is integrated in chromosome 2 p23.2 (unpublished). (A complete analysis of the integration site sequence will be published at a later date.) The viral splice donor at nucleotide 880 is fused to an intergenic cryptic acceptor. There are no obvious genes encoded in the vicinity of this region that could account for the high occupancy of super-enhancer markers in the integrated viral DNA. To ensure that the sensitivity of W12 20861 cells to the bromodomain inhibitors was due to downregulation of E6 and E7, and not due to downregulation of cellular oncogenes driven by either the HPV16 super-enhancer-like element or another Brd4-dependent super-enhancer, we analyzed gene expression of 20861 and 20863 cells after treatment with iBET72+. Genes that were upregulated and downregulated in response to iBET treatment in 20861 versus 20863 cells are shown in Table S1 and Fig. S5 in the supplemental material. In general, the vast majority of genes showed a similar response in both cell lines. The few genes that had notable difference in magnitude of gene expression changes are listed in Table S2 and have no obvious connection to cellular proliferation. Therefore, the responses of 20861 and 20863 to BET inhibitors are similar, and growth suppression is primarily due to downregulation of E6 and E7. It is well characterized that downregulation of E6 and E7 expression in cells dependent on their growth results in p21^Cip1^-dependent growth arrest and senescence, and our findings mirror these observations (35–37). A comparison of expression changes in senescence-related genes identified by Wells et al. (35) is shown in Table S3 in the supplemental material. Although these data cannot show that the BET inhibitors directly and solely inhibit proliferation through downregulation of the E6 and E7 viral proteins, taken together with studies of others (35–37), it seems likely. Also, in conclusion, while we had hypothesized that the growth of 20861 cells would be more sensitive to inhibition of Brd4 binding than 20863 cells, it seems that any cell line that has become addicted to E6 and E7 oncogene function is exquisitely sensitive to their downregulation.

## DISCUSSION

We have shown that the tandemly repeated HPV16 integration locus in W12 20861 cells has properties similar to those described for super-enhancers. The chromatin at this locus is so highly enriched in the super-enhancer markers Brd4, MED1, and H3K27ac, that they are observed as a very prominent nuclear domain by indirect immunofluorescence. HPV cancer cells are “addicted” to HPV E6/E7 oncogene expression, and downregulation of these proteins results in reduction of cellular proliferation and induction of senescence (33, 36). Treatment of 20861 cells with acetyl-lysine histone mimics disrupts Brd4 binding, resulting in decreased HPV16 E6 and E7 transcription and induction of senescence.

The role of Brd4 in regulating E6/E7 transcription has been well studied, but most investigations have focused on the ability of E2 to repress the major early promoter in a Brd4-dependent manner (13, 14, 16, 38). E2 recruits Brd4 via the E2 binding sites adjacent to the viral promoter and inhibits transcription, in part because E2 binding to the C terminus of E2 competes with Brd4 recruitment of pTEFb (16). In W12 20861 cells, we show clearly that Brd4 activates transcription from the integrated HPV16 P_97_ promoter. Similarly, it has been shown that Brd4 activates the viral early promoter in integrated HPV (in the absence of E2) in both HeLa and Caski cells (16). Notably, in our study Brd4 also activates transcription from the P_97_ promoter in the extrachromosomal HPV genomes in 20863 cells, even though E2 is assumed to be present (39). These findings are consistent with those of Helfer et al. (40), who also showed that iBET inhibitors reduced HPV transcription in 20863 cells. Bechtold et al. have demonstrated that the P_97_ promoter in the extrachromosomal HPV16 genomes in 20863 cells is impervious to repression by E2, and the authors hypothesize that chromatin renders the promoter inaccessible to E2 binding (39). We also show that disruption of Brd4 binding downregulates E6-E7 transcription and results in growth repression/cell death. As shown by others, and confirmed here by gene expression analysis, this is most likely due to reactivation of the p53 and Rb tumor suppressor pathways leading to induction of irreversible cellular senescence mediated by p21^Cip1^ (35–37). Both 20861 and 20863 cells were sensitive to treatment with BET inhibitors, showing that Brd4 can activate transcription of both integrated and extrachromosomal HPV16 genomes. It has been shown that E2 expression does not inhibit E6/E7 expression in 20863 extrachromosomal genomes (39) and is usually not expressed from integrated genomes.

Both 20861 and 20863 cells are sensitive to treatment with BET inhibitors, and viral transcription is dramatically downregulated in both cases. Furthermore, both integrated and extrachromosomal HPV genomes are enriched in Brd4, MED1, and H3K27ac, so what is the significance of the HPV16 super-enhancer-like element? Super-enhancers are defined by the high intensity of these factors, not just their presence at enhancers. 20861 cells express much higher levels of E7 and have increased plating efficiency and a growth advantage compared to 20863 cells (2). However, although the super-enhancer-like element drives very high levels of E6 and E7, the BET inhibitors efficiently disrupt E6/E7 transcription in both cases, leading to cellular senescence. Almost all HPV-containing keratinocyte cells are completely dependent on the expression of E6 and E7, and therefore it would be expected that BET inhibitors could reduce E6/E7 expression (if transcription was dependent on Brd4) and result in growth suppression. Further studies are essential to determine how often Brd4 activates viral transcription in HPV infections and cancers, and if this is widespread, iBET inhibitors could be therapeutic for a wide range of HPV-associated diseases. A very recent study by Groves et al. (using a different series of W12 subclones and with HPV integrated at different sites than 20861 cells) also showed that E6 and E7 expression levels correlate with the balance of active chromatin modifications at the HPV integration site (41). However, as shown here, the prominence of the Brd4 nuclear focus in the 20861 subclone is somewhat unusual. Kalantari and colleagues demonstrated that, compared to other subclones, the integrated HPV16 genomes in 20861 cells were notable for particularly high levels of E6 and E7 expression and absence of DNA methylation (42). In contrast, many other cells such as the well-studied cell line Caski, are highly methylated and only the 3′-junctional copy of the viral genome is transcriptionally active (3). Cellular super-enhancers have also been shown to form in DNA methylation valleys (DMVs) (18). We hypothesize that the tandem copies of unmethylated URR in 20861 cells create a high density of binding sites for transcription activation complexes that include Brd4 and MED1; these act as a super-enhancer-like element for viral transcription from the promoter at the virus-cell junction copy. Thus, the ability of integrated HPV to act as a super-enhancer for viral transcription may depend on the copy number and/or methylation status of the integrated copies. It is becoming apparent that each HPV integration site has unique characteristics that depend on viral and cellular breakpoints, rearrangement, and amplification of viral and cellular junctional sequences and epigenetic regulation (41, 43). Furthermore, the organization of integration sites can be dynamic and driven by selection for increased viral oncogene expression (43).

## MATERIALS AND METHODS

### Cell culture.

Keratinocyte cell lines were cultured in F medium (3:1 [vol/vol] F-12–Dulbecco’s modified Eagle’s medium [DMEM], 5% fetal bovine serum [FBS], 0.4 µg/ml hydrocortisone, 5 µg/ml insulin, 8.4 ng/ml cholera toxin, 10 ng/ml epidermal growth factor [EGF], 24 µg/ml adenine, 100 U/ml penicillin, and 100 µg/ml streptomycin); 1a keratinocytes were supplemented with 10 µM Y-27632 (44). All cells were grown in the presence of irradiated 3T3-J2 feeder cells. The W12-derived subclones 9E and 6E were described previously (2, 45).

### BET inhibitors.

iBET762+ and iBET762− were synthesized as described previously (26) or purchased from BioVision. JQ1+ and JQ1− are from BioVision.

### siRNA.

Brd4 siRNA (Hs_BRD4_6 FlexiTube, SI03190845; Qiagen) or control siRNA (AllStars negative control, 1027281; Qiagen) was transfected into 20861 cells using RNAimax, following the manufacturer’s instruction. The following antibodies were used: ant-γ-H2AX (phospho-histone H2AX [Ser139], 05-636 [Millipore]), anti-Rad51 (ab213 [Abcam]), anti-MED1 (A300-793A [Bethyl Laboratories]), anti-H3K27ac (07-360 [Millipore]), anti-CDK9 (sc-8338 [Santa Cruz]), and anti-Brd4 (A301-985A; 1.2 µg per IP [Bethyl Laboratories]). Affinity-purified Brd4 C-terminus-specific anti-Brd4 antiserum (MCB2) has been described previously (12). Brd4 (8H2 mouse monoclonal antibody) binds the BDII region and is from Cheng-Ming Chiang (UT Southwestern) (46). For immunofluorescence, antibodies were diluted 1:100. For ChIP, 3.0 µg antibody was used per IP unless stated otherwise.

### ChIP.

For chromatin immunoprecipitation (ChIP), W12 cells were fixed in 1% formaldehyde for 10 min at room temperature, quenched with 125 mM glycine for 5 min at room temperature, washed twice with ice-cold phosphate-buffered saline (PBS), and pelleted for 10 min at 300 × *g* at 4°C. Cell pellets were resuspended in 1 ml lysis buffer I (50 mM HEPES KOH [pH 7.5], 140 mM NaCl, 1 mM EDTA [pH 8.0], 10% glycerol, 0.5% NP-40, 0. 5% Triton X-100, Complete protease inhibitor cocktail), homogenized using a FastPrep-24 tissue homogenizer at 3 cycles of 20-s pulses at 6.0 m/s with 2-min incubations on ice in between cycles, resuspended in 1 ml lysis buffer II (10 mM Tris-Hl [pH 8.0], 200 mM NaCl, 1 mM EDTA [pH 8.0], 0.5 mM EGTA [pH 8.0], Complete protease inhibitor cocktail) for 10 min at 4°C, and then resuspended in lysis buffer III (50 mM Tris-HCl [pH 8.0], 10 mM EDTA [pH 8.0], 1% SDS, Complete protease inhibitor cocktail). Chromatin was sheared into DNA fragments ranging between 200 and 800 bp using a Bioruptor sonicator (Diagonode) on high-power settings, supplemented with 1% Triton X-100 solution, and centrifuged for 10 min at 16,000 × *g* at 4°C to remove cellular debris. Chromatin samples (20 µg per ChIP) were incubated overnight at 4°C with antibodies against Brd4, H3K27ac, and MED1. No-antibody controls were included to measure nonspecific binding. To precipitate the chromatin immunocomplexes, 50 µl blocked Dynabeads with protein G (Invitrogen) was added to each sample and incubated for 1 h at 4°C. Immunoprecipitates were washed once each with 1 ml low-salt wash buffer (20 mM Tris-HCl [pH 8.0], 150 mM NaCl, 2 mM EDTA [pH 8.0], 1% Triton X-100, 0.1% SDS), 1-ml high-salt wash buffer (20 mM Tris-HCl [pH 8.0], 500 mM NaCl, 2 mM EDTA [pH 8.0], 1% Triton X-100, 0.1% SDS), and 1 ml LiCl wash buffer (10 mM Tris-HCl [pH 8.0], 250 mM LiCl, 1 mM EDTA [pH 8.0], 1% IGEPAL, 1% sodium deoxycholate) and twice with 1-ml Tris-EDTA (TE) buffer. Chromatin was eluted using 100 µl elution buffer (50 mM Tris-HCl [pH 8.0], 10 mM EDTA [pH 8.0], 1% SDS) and incubated with shaking for 30 min at 65°C. Eluted chromatin DNA was reverse cross-linked overnight at 65°C with 5 M NaCl, followed by RNase A and proteinase K treatment, and purified using the ChIP DNA Clean and Concentrator kit (Zymo Research). ChIP DNA was analyzed by real-time qPCR.

### RNA preparation.

Total cellular RNA was extracted using an RNeasy minikit (Qiagen). RNA integrity was verified using the Bioanalyzer 2100 (Agilent Technologies). One microgram of total cellular RNA was reverse transcribed using a Transcriptor first-strand cDNA kit (Roche).

### Real-time qPCR.

Real-time quantitative PCR (qPCR) was performed using the ABI Prism 7900HT sequence detector (Applied Biosystems) and SYBR green PCR master mix (Applied Biosystems). All reactions were run in triplicate and compared to standard curves of cloned HPV16 E6*I cDNAs (for gene expression profiling), input chromatin DNA (for ChIP assays), or cloned HPV16 genome from W12 cells (for determination of viral copy number), to generate a standard curve of threshold cycle (*C_T_*) versus log_10_ quantity (picograms). The primers used for gene expression profiling were as follows: E6*I, sense (n208–226^409–411), 5′-ACAGTTACTGCGACGTGAGGTG-3′, and antisense (n445–428), 5′-TTCTTCAGGACACAGTGG-3′; and Brd4, sense, 5′-ACCAGTTTGCATGGCCTTTC-3′, and antisense, 5′-AATGATCTTATAGTAATCAGGGAGGTTCA-3′. (The primers for Brd4 span exons 2 to 3 targeting all transcripts.) All values were normalized to levels of 28S rRNA (sense, 5′-AGTCGGGTTGCTTGGGAATGC-3′; antisense, 5′-CCCTTACGGTACTTGTTGACT-3′) or PPIA (sense, 5′-AGAACTTCATCCTAAAGCATACGG-3′; antisense, 5′-TGCTTGCCATCCAACCACTC-3′). The primers used for ChIP were as follows: URR (n7459–7556), sense, 5′-TTCGGTTGCATGCTTTTTGGCACAA-3′, and antisense, 5′-CACGCATGGCAAGCAGGAAACGTAC-3′; L1 (n5649–5728), sense, 5′-GGCTGCCTAGTGAGGCCACTG-3′, and antisense, 5′-GCGTGCAACATATTCATCCGTGC-3′; E2 (n3412–3505), sense, 5′-CGCCGCGACCCATACCAAAG-3′, and antisense, 5′-GGGGTTTCCGGTGTCTGGCT-3′; CCND2 5′ untranslated region (UTR) (chr12:4383036–4383111), sense, 5′-CCTTCTGCTCCACCTTCTCT-3′, and antisense, 5′-CTGACCTCCTTCCTTTGGCT-3′; FOSL1 intron 1 (chr11:65666566–65666657), sense, 5′-AACGTCCCTGTTCCCATTCT-3′, and antisense, 5′-GGCAGAGCTAGAACCCACTT-3′; and IGLL5 downstream region (chr22:23272085–23272161), sense, 5′-CTTGAGGATTGCAGATGGGC-3′, and antisense, 5′-TGACCCTGATCCTGACCCTA-3′. The data were analyzed with SDS 2.1 software (Applied Biosystems).

### IF.

For immunofluorescence (IF) analysis, cells were cultured on coverslips and fixed at room temperature in 4% paraformaldehyde (PFA)–PBS for 20 min. Cells were permeabilized in 0.1% Triton X-100 and stained with primary and fluorescent secondary antibodies using standard procedures. Coverslips were mounted in ProLong Gold containing 4′,6-diamidino-2-phenylindole (DAPI) for analysis by confocal microscopy.

### IF-FISH.

Cells grown on coverslips were fixed in cold methanol-acetic acid (3:1) for 3 min and 4% paraformaldehyde–PBS for 20 min. Immunofluorescence was performed as described above. Antibodies were fixed *in situ* with methanol-acetic acid (3:1) at room temperature for 10 min and 2% paraformaldehyde–PBS at room temperature for 2 min. Cells were treated with RNase A and dehydrated in a 70%, 90%, and 100% ethanol series for 3 min each. Full-length HPV16 DNA was fluorescently labeled using the Ulysis nucleic acid labeling kit (Life Technologies). Seventy-five to 360 ng labeled fluorescence *in situ* hybridization (FISH) probe in hybridization buffer (Empire Genomics) was added to the coverslip, and DNA was denatured at 75°C for 5 min, followed by hybridization at 37°C overnight. Cells were washed with 1× phosphate-buffered detergent (Qbiogene) for 5 min at room temperature, 1× wash buffer (0.5× SSC, 0.1% SDS) for 5 min at 65°C, and 1× phosphate-buffered detergent (Qbiogene) for 5 min at room temperature. Coverslips were mounted in ProLong Gold containing DAPI for analysis by confocal microscopy.

### Confocal microscopy and image analysis.

Images were collected using a Leica TCS-SP5 laser scanning confocal imaging system and processed using Leica, Las AF Lite software. Data and images shown were obtained from z-stacks of 10 slices encompassing the entire cell and combined using maximum projection, or from single optical slices (as indicated). Colocalization was determined using Leica Microsystems Las AF Lite software. Single z-stack images were taken at the foci. A region of interest line (shown in white) was drawn simultaneously in all channels (blue, red, and green) so that it spanned the nucleus and intersected the foci (Fig. 4). Data of relative staining intensity versus distance was exported and processed using Microsoft Excel.

### Microarray expression analysis.

Gene expression analysis was carried out in the Research Technologies Branch, NIAID. RNA integrity was verified using a Bioanalyzer. Five hundred nanograms of RNA was amplified and labeled using the Illumina TotalPrep RNA amplification kit (Applied Biosystems), hybridized to Illumina HumanHT-12 v4 Expression BeadChip, and scanned using the Illumina HiScan-SQ. Signal data were extracted from image files with the Gene Expression module (v.1.9.0) of the GenomeStudio software (v.2011.1) from Illumina, Inc. Signal intensities were converted to log_2_ scale. Analysis of variance (ANOVA) was performed on the normalized signals to test mRNA expression differences between 20861 and 20863 cells treated with iBET72+ and iBET72−. Significance was determined by a false discovery rate at 0.05 to account for multiple comparisons. Statistical analysis was performed primarily in JMP/Genomics 7.0 (SAS Institute Inc., Cary, NC).

### Accession number(s).

All data have been uploaded to GEO under accession no. GSE75987.

## SUPPLEMENTAL MATERIAL

Figure S1  The prominent Brd4 focus does not colocalize with Rad51 or γ-H2AX in 20861 cells. 20861 cells were costained for Brd4 (C-terminal antibody), γ-H2AX, and Rad51, which are markers for DNA damage and recombination. Cells were inspected for a prominent Brd4 focus; a minimum of 220 cells were examined per experiment for colocalization with Rad51 and γ-H2AX. Colocalization events were rare: 4% ± 2% of Brd4 foci colocalized with γ-H2AX, and 3% ± 1% of Brd4 foci colocalized with Rad51 (mean + SD; *n =* 3). The dotted line outlines the nucleus as detected by DAPI staining. Images are from a single optical slice. Download Figure S1, PDF file, 1 MB

Figure S2  Brd4 foci do not persist throughout mitosis in 20861 cells. (A) Mitotic cells stained with antibodies to BDII region of Brd4 (green) and H3K27ac (red) were analyzed for a prominent speckle on chromosomes. (B) 20861 cells were analyzed for Brd4 and H3K27ac foci at each stage of mitosis in three independent experiments (8 to 22 cells per mitotic phase in each experiment). Both the numbers of Brd4 foci (shown here) and intensity of signal (data not shown) decreased considerably in mid-mitosis. Images are from a single optical slice. (C) IF-FISH of interphase and mitotic cells stained with antibody to Brd4 (McB2 [red]) and hybridized with HPV16 DNA (green). The arrows show the HPV16 integration locus. Download Figure S2, PDF file, 16 MB

Figure S3  siRNA depletion of Brd4 downregulates HPV16 viral transcription in 20861 cells. 20861 cells were transfected with 20 nM siRNA negative control (siCtrl) or 20 nM siRNA targeting Brd4 (siBrd4) for 72 h, and Brd4 (A) and HPV16 E6*I (B) transcription levels were determined by real-time qPCR. Absolute quantification of Brd4 and E6*I cDNA was determined using a standard curve and normalized to PPIA. Results represent three independent experiments. Error bars represent SD. An unpaired Student’s *t* test was used to determine statistical significance between treatments. **, *P* < 0.01. Download Figure S3, PDF file, 0.2 MB

Figure S4  Proliferation of 20861 cells is inhibited by disruption of Brd4 binding. (A) C-33A, 20861, and 20863 cells were plated at low density, and proliferation was measured (as percentage of confluence) with an Incucyte microscope (Essen Biosciences). After 12 h of measurement (arrow), JQ1− (negative-control stereoisomer) or JQ1+ was added to the medium at a final concentration of 0, 4, 8, 12, 16, or 25 nM, and growth was measured for a total of 112 h. (B) At the end of the growth period, cells were fixed and stained with methylene blue. Five replicate growth curves were measured for 20861 and 20863 and three for C-33A. A representative experiment is shown. Download Figure S4, PDF file, 18.3 MB

Figure S5  Bivariate fit of estimate of effect of iBET72− or iBET72+ on 20861 and 20863 cells. Shown is a comparison of the effects of iBET72− or iBET72+ on 20861 and 20863 cells. Difference between treatment groups is shown in the log_2_ scale. Values for individual genes are shown in gray. Values for genes located on chromosome 2 (which contains integrated HPV16 locus) are shown in black. Linear fit analysis was performed in JMP v.12.0.1 (SAS Institutes). Linear fit represents estimate of iBET72+ effect on 20861 = 9.434e−6 + 0.9824377 × estimate of iBET72+ effect on 20863. *R*^2^ = 0.685992. Download Figure S5, PDF file, 0.3 MB

Table S1  Genes regulated by iBET72 in 20861 and 20863 cells. Shown are genes with more than 2-fold changes in expression after iBET72 treatment (*P* < 0.05 in 20861 cells). Table S1, XLSX file, 0.1 MB

Table S2  Differential gene expression between 20861 and 20863 cells after iBET72 treatment. Shown are genes with more than 2-fold changes in expression after iBET72 treatment in 20861 cells (*P* < 0.05) but with no significant change in 20863 cells (*P* < 0.05). Table S2, XLSX file, 0.02 MB

Table S3  Genes associated with senescence. Shown is a comparison with genes described by Wells et al. (35). Listed are genes in our study significantly changed by iBET72+ treatment in 20861 cells (*P* ≤ 0.05), which were also shown to be changed in the study by Wells et al., where E6 and E7 gene expression was repressed in HeLa cells by E2 expression. Table S3, XLSX file, 0.02 MB
