# Supplementary material for: Tandemly Integrated HPV16 Can Form a Brd4-Dependent Super-Enhancer-Like Element That Drives Transcription of Viral Oncogenes
Source: mBio. 2016 Sep 13;7(5):e01446-16. doi: 10.1128/mBio.01446-16 (PMC5021809; doi:10.1128/mBio.01446-16)
Supplement: Figure S1 — The prominent Brd4 focus does not colocalize with Rad51 or γ-H2AX in 20861 cells. 20861 cells were costained for Brd4 (C-terminal antibody), γ-H2AX, and Rad51, which are markers for DNA damage and recombination. Cells were inspected for a prominent Brd4 focus; a minimum of 220 cells were examined per experiment for colocalization with Rad51 and γ-H2AX. Colocalization events were rare: 4% ± 2% of Brd4 foci colocalized with γ-H2AX, and 3% ± 1% of Brd4 foci colocalized with Rad51 (mean + SD; n = 3). The dotted line outlines the nucleus as detected by DAPI staining. Images are from a single optical slice. Download [file mbo004162981sf1.pdf]

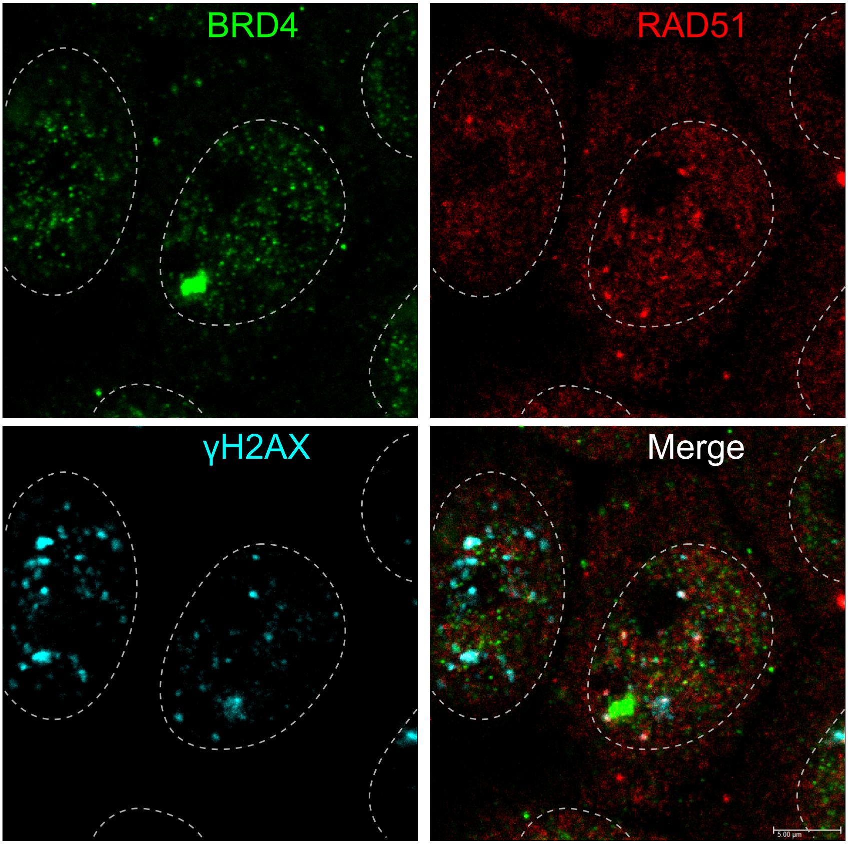

Supplementary Figure 1: The prominent BRD4 focus does not colocalize with RAD51 or γ-H2AX in 20861 cells

20861 cells were co-stained for BRD4 (C-terminal antibody), γH2AX, and RAD51, which are markers for DNA damage and recombination. Cells were inspected for a prominent BRD4 focus; a minimum of 220 cells were examined per experiment for co-localization with RAD51 and γ-H2AX. Colocalization events were rare: 4+2% of BRD4 foci colocalized with γ-H2AX and 3+1% BRD4 foci colocalized with RAD51 (+SD, N=3). The dotted line outlines the nucleus as detected by DAPI staining. Images are from a single optical slice.
