# Supplementary material for: Tandemly Integrated HPV16 Can Form a Brd4-Dependent Super-Enhancer-Like Element That Drives Transcription of Viral Oncogenes
Source: mBio. 2016 Sep 13;7(5):e01446-16. doi: 10.1128/mBio.01446-16 (PMC5021809; doi:10.1128/mBio.01446-16)
Supplement: Figure S2 — Brd4 foci do not persist throughout mitosis in 20861 cells. (A) Mitotic cells stained with antibodies to BDII region of Brd4 (green) and H3K27ac (red) were analyzed for a prominent speckle on chromosomes. (B) 20861 cells were analyzed for Brd4 and H3K27ac foci at each stage of mitosis in three independent experiments (8 to 22 cells per mitotic phase in each experiment). Both the numbers of Brd4 foci (shown here) and intensity of signal (data not shown) decreased considerably in mid-mitosis. Images are from a single optical slice. (C) IF-FISH of interphase and mitotic cells stained with antibody to Brd4 (McB2 [red]) and hybridized with HPV16 DNA (green). The arrows show the HPV16 integration locus. Download [file mbo004162981sf2.pdf]

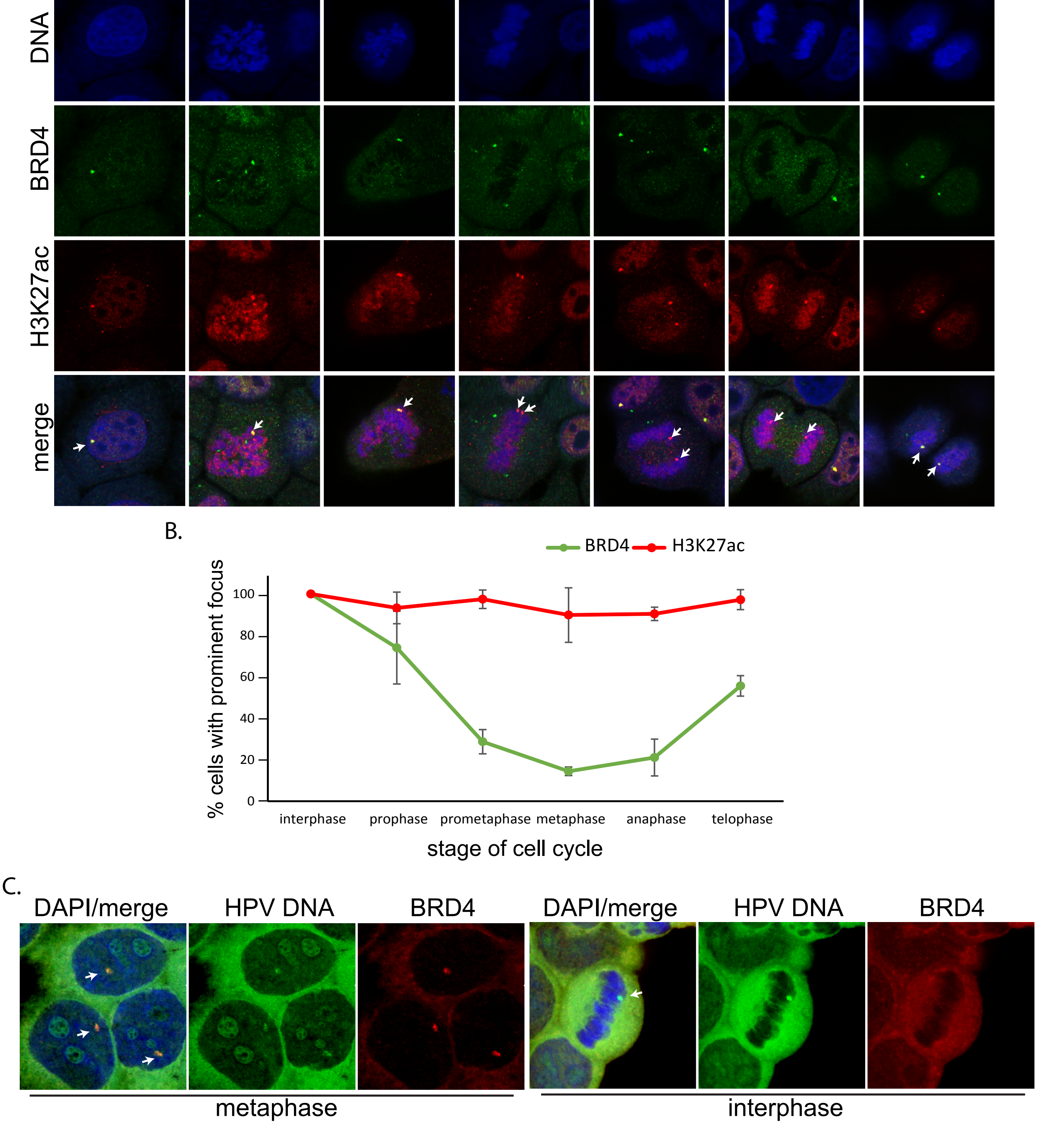

Supplementary Figure 2: BRD4 foci do not persist throughout mitosis in 20861 cells
A. Mitotic cells stained with antibodies to BDII region of BRD4 (green) and H3K27ac (red) were analyzed for a prominent speckle on chromosomes. The arrows show the H3K27ac focus, which persists throughout mitosis. B. 20861 cells were analyzed for BRD4 and H3K27ac foci at each stage of mitosis in three independent experiments (8-22 cells per mitotic phase in each experiment). Both the numbers of BRD4 foci (shown here) and intensity of signal (data not shown) decreased considerably in mid-mitosis. Images are from a single optical slice. C. IF-FISH of interphase and mitotic cells stained with antibody to Brd4 (McB2; red) and hybridized with HPV16 DNA (green). The arrows show the HPV16 integration locus.
