# Supplementary material for: Tandemly Integrated HPV16 Can Form a Brd4-Dependent Super-Enhancer-Like Element That Drives Transcription of Viral Oncogenes
Source: mBio. 2016 Sep 13;7(5):e01446-16. doi: 10.1128/mBio.01446-16 (PMC5021809; doi:10.1128/mBio.01446-16)
Supplement: Figure S3 — siRNA depletion of Brd4 downregulates HPV16 viral transcription in 20861 cells. 20861 cells were transfected with 20 nM siRNA negative control (siCtrl) or 20 nM siRNA targeting Brd4 (siBrd4) for 72 h, and Brd4 (A) and HPV16 E6*I (B) transcription levels were determined by real-time qPCR. Absolute quantification of Brd4 and E6*I cDNA was determined using a standard curve and normalized to PPIA. Results represent three independent experiments. Error bars represent SD. An unpaired Student’s t test was used to determine statistical significance between treatments. **, P < 0.01. Download [file mbo004162981sf3.pdf]

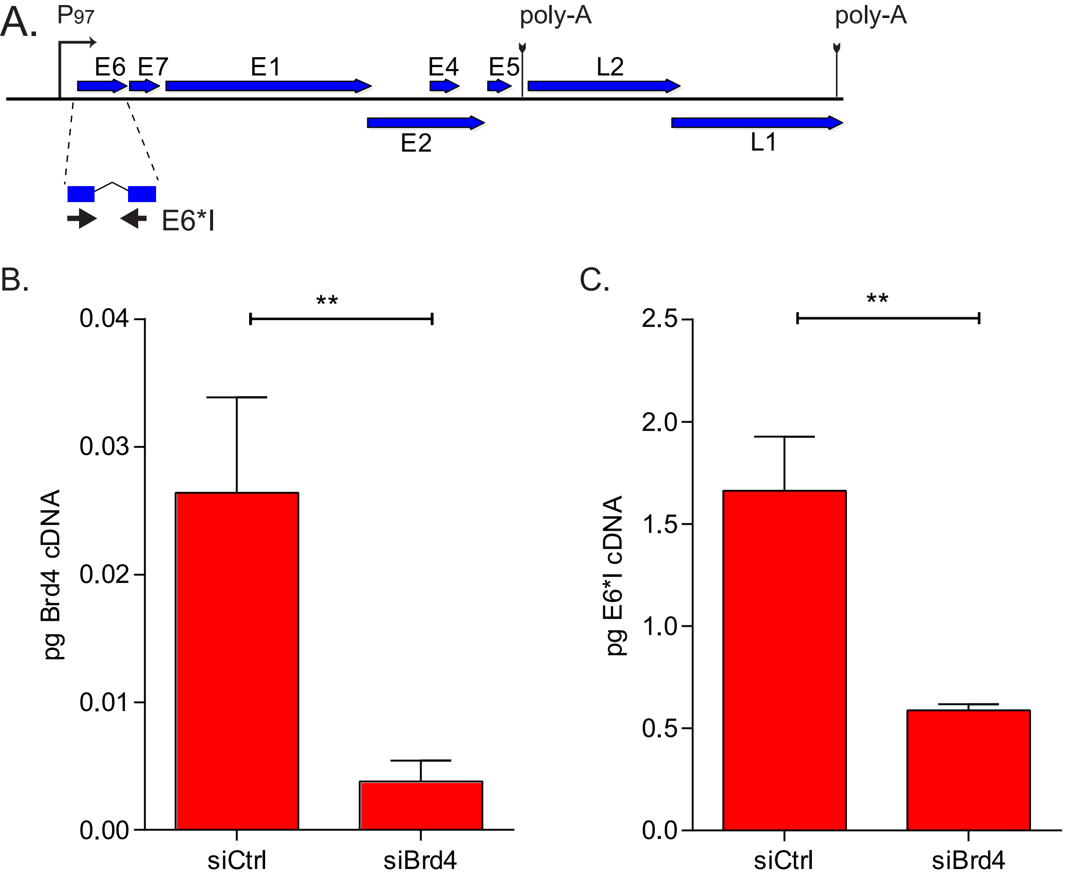

Supplementary Figure 3:
siRNA depletion of Brd4 downregulates HPV16 viral transcription in 20861 cells

A. Map of linearized HPV16 genome with position of the E6*I transcript and location of primers used to detect E6*I cDNA.
B and C. 20861 cells were transfected with 20 nM siRNA negative control (siCtrl) or 20 nM siRNA targeting Brd4 (siBrd4) for 72 hours and Brd4 (B) and HPV16 E6*I (C) transcription levels determined by real-time RT-qPCR. Absolute quantification of Brd4 and E6*I cDNA was determined using a standard curve and normalized to PPIA. Results represent three independent experiments. Error bars represent SD. An unpaired Student's t-test was used to determine statistical significance between treatments; **$p < 0.01$.
