# Supplementary material for: Tandemly Integrated HPV16 Can Form a Brd4-Dependent Super-Enhancer-Like Element That Drives Transcription of Viral Oncogenes
Source: mBio. 2016 Sep 13;7(5):e01446-16. doi: 10.1128/mBio.01446-16 (PMC5021809; doi:10.1128/mBio.01446-16)
Supplement: Figure S4 — Proliferation of 20861 cells is inhibited by disruption of Brd4 binding. (A) C-33A, 20861, and 20863 cells were plated at low density, and proliferation was measured (as percentage of confluence) with an Incucyte microscope (Essen Biosciences). After 12 h of measurement (arrow), JQ1− (negative-control stereoisomer) or JQ1+ was added to the medium at a final concentration of 0, 4, 8, 12, 16, or 25 nM, and growth was measured for a total of 112 h. (B) At the end of the growth period, cells were fixed and stained with methylene blue. Five replicate growth curves were measured for 20861 and 20863 and three for C-33A. A representative experiment is shown. Download [file mbo004162981sf4.pdf]

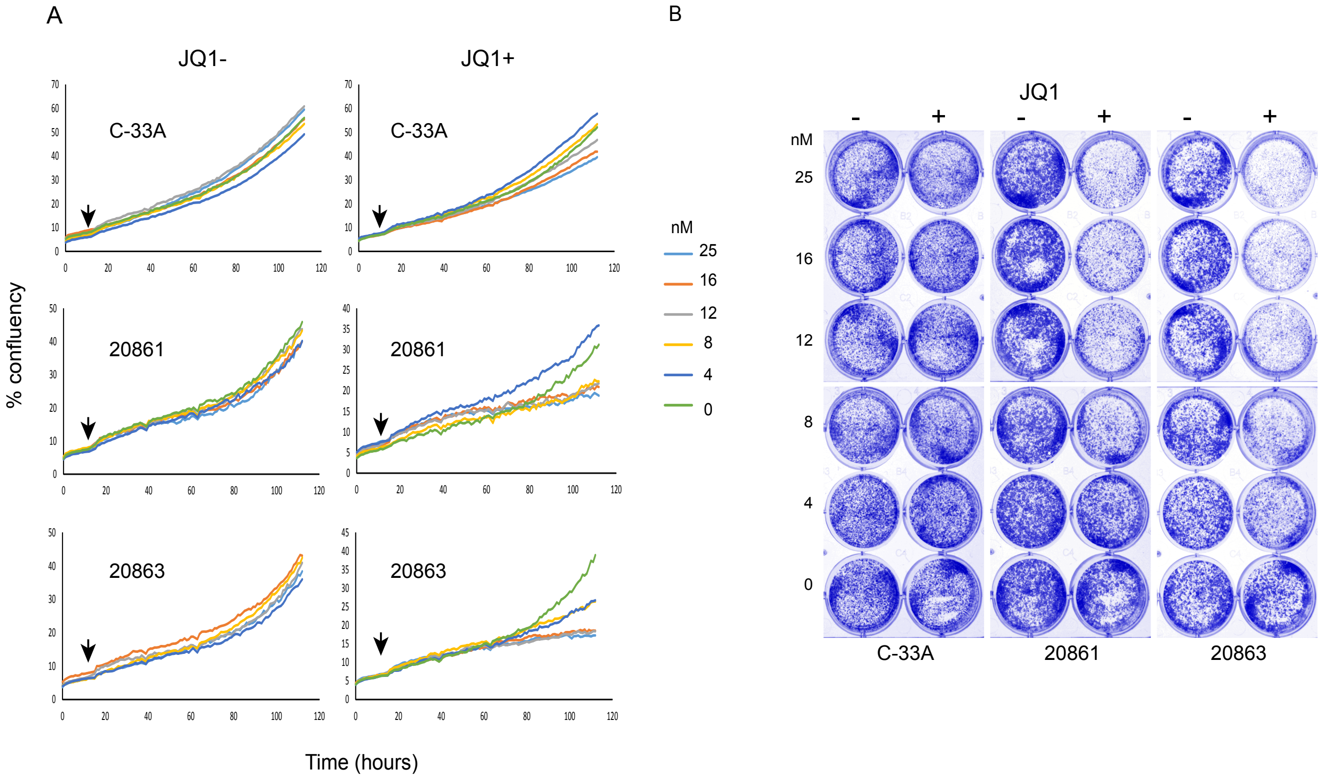

Supplementary Figure 4: 20861 cell proliferation is inhibited by disruption of BRD4 binding

A. C-33A, 20861 and 20863 cells were plated at low density and proliferation was measured (as % confluence) with an Incucyte microscope (Essen Biosciences). After 12 hours of measurement JQ1- (negative control stereoisomer) or JQ1+ was added to the medium (time point represented by black arrow) at a final concentration of 0, 4, 8, 12, 16 or 25nM and growth was measured for a total of 112 hours. B. At the end of the growing period, cells were fixed and stained with methylene blue. Five independent replicate growth curves were measured for 20861 and 20863, and three for C-33A. A representative experiment is shown.
