# Supplementary material for: Tandemly Integrated HPV16 Can Form a Brd4-Dependent Super-Enhancer-Like Element That Drives Transcription of Viral Oncogenes
Source: mBio. 2016 Sep 13;7(5):e01446-16. doi: 10.1128/mBio.01446-16 (PMC5021809; doi:10.1128/mBio.01446-16)
Supplement: Figure S5 — Bivariate fit of estimate of effect of iBET72− or iBET72+ on 20861 and 20863 cells. Shown is a comparison of the effects of iBET72− or iBET72+ on 20861 and 20863 cells. Difference between treatment groups is shown in the log2 scale. Values for individual genes are shown in gray. Values for genes located on chromosome 2 (which contains integrated HPV16 locus) are shown in black. Linear fit analysis was performed in JMP v.12.0.1 (SAS Institutes). Linear fit represents estimate of iBET72+ effect on 20861 = 9.434e−6 + 0.9824377 × estimate of iBET72+ effect on 20863. R2 = 0.685992. Download [file mbo004162981sf5.pdf]

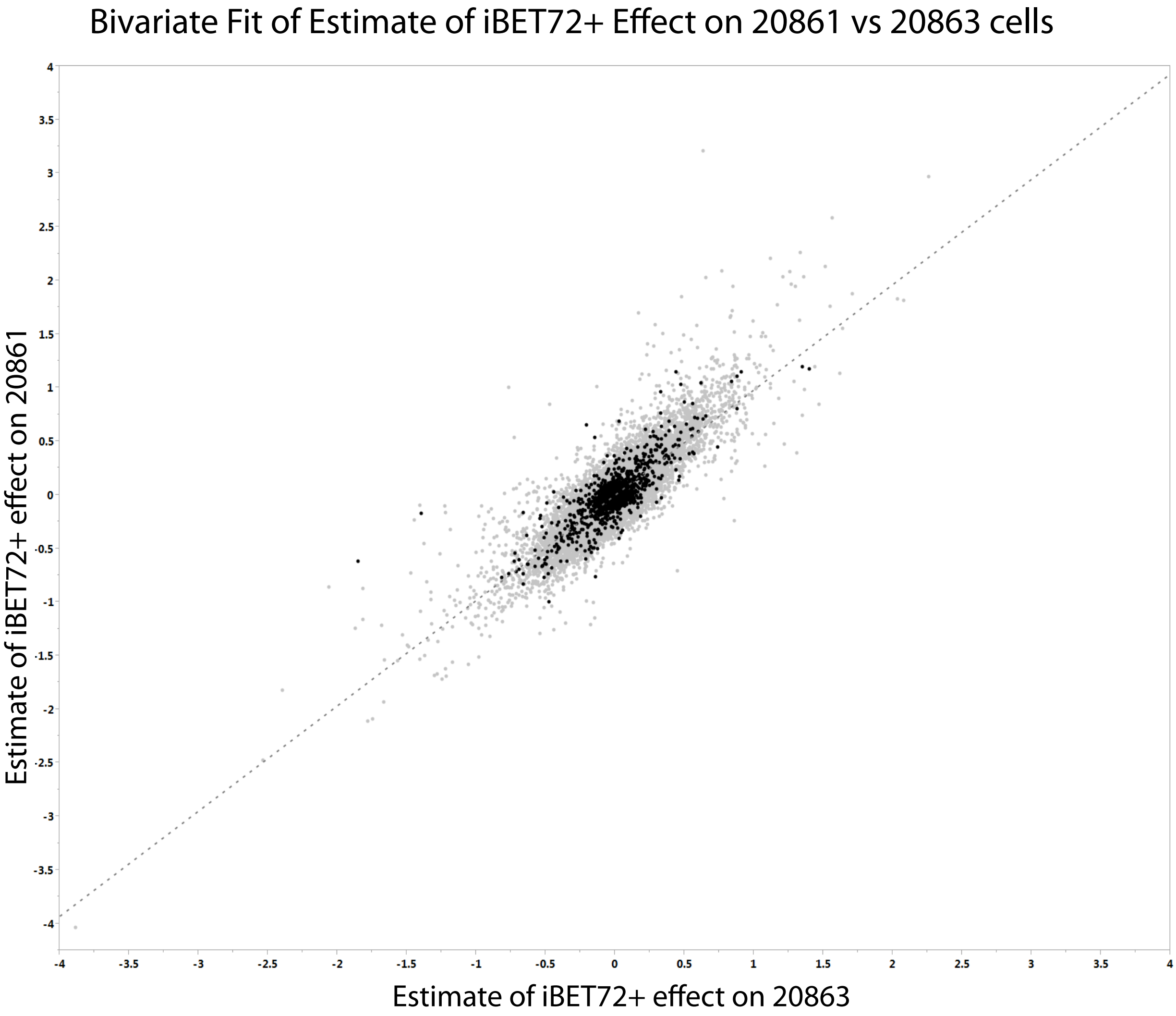

Supplementary Figure 5 Comparison of effect of iBET72- or iBET72+ effect on 20861 and 20863 cells.
Difference between treatment groups is log2 scale. Values for individual genes are shown in grey. Values for genes located on chromosome 2 (which contains integrated HPV16 locus) are shown in black. Linear fit analysis was performed in JMP v 12.0.1 (SAS Institutes). Genes with more than two-fold changes in expression after iBET72 treatment in 20861 cells ($P<0.05$), but with no significant change in 20863 cells ($P<0.05$) are shown in Supplementary Table 1
Linear fit: Estimate of iBET72+ effect on 20861=9.434e-6 + 0.9824377*Estimate of iBET72+ effect on 20863 Rsquare=0.685992
